# Pre-existing Opioid Use Worsens Outcomes in Patients With Diverticulitis

**DOI:** 10.7759/cureus.34624

**Published:** 2023-02-04

**Authors:** Amjad Shaikh, Ayham Khrais, Alexander Le, Alexander J Kaye, Sushil Ahlawat

**Affiliations:** 1 Department of Medicine, Rutgers University New Jersey Medical School, Newark, USA; 2 Department of Gastroenterology and Hepatology, Rutgers University New Jersey Medical School, Newark, USA

**Keywords:** colon abscess, substance use disorder (sud), diverticular disease of the colon, opioid use, diverticulitis colon

## Abstract

Background and objective

Diverticulitis occurs in 10-25% of patients with diverticulosis. Although opioids can decrease bowel motility, there is scarce data on the effect of chronic opioid use on the outcomes of diverticulitis. In this study, we aimed to explore the outcomes of diverticulitis in patients with pre-existing opioid use.

Methods

Data between 2008 and 2014 from the National Inpatient Sample (NIS) database was extracted using the International Classification of Diseases, 9th Revision (ICD-9) codes. Univariate and multivariate analyses were used to generate odds ratios (OR). Elixhauser Comorbidity Index (ECI) scores predicting mortality and readmission were calculated based on weighted scores from 29 different comorbidities. Scores were compared between the two groups using univariate analysis. Inclusion criteria included patients with a primary diagnosis of diverticulitis. Exclusion criteria included patients less than 18 years of age, and a diagnosis of opioid use disorder in remission. Studied outcomes included inpatient mortality, complications (including perforation, bleeding, sepsis event, ileus, abscess, obstruction, and fistula), length of hospital stay, and total costs.

Results

A total of 151,708 patients with diverticulitis and no active opioid use and 2,980 patients with diverticulitis and active opioid use were hospitalized in the United States from 2008 to 2014. Opioid users had a higher OR for bleeding, sepsis, obstruction, and fistula formation. Opioid users had a lower risk of developing abscesses. They had longer lengths of stay, higher total hospital charges, and higher Elixhauser readmission scores.

Conclusion

Hospitalized diverticulitis patients with comorbid opioid use are at an elevated risk of in-hospital mortality and sepsis. This could be attributed to complications from injection drug use predisposing opioid users to these risk factors. Outpatient providers caring for patients with diverticulosis should consider screening their patients for opioid use and try offering them medication-assisted treatment to reduce their risk of poor outcomes.

## Introduction

Diverticulosis is a highly prevalent disease, affecting nearly 180 out of 100,000 people yearly, and its incidence has been on the rise [1-2]. Its pathophysiology involves the development of elevated intraluminal pressure, affecting the weakest points of the colonic wall bisected by the vasa recta [3-5]. Disruption of the colon’s structural integrity at these sites results in focal outpouchings, thereby forming diverticula [4]. Inflammation of these diverticula, which is termed diverticulitis, emanates from further increases in intraluminal pressure, resulting in colonic microperforation [4]. In effect, diverticular disease is associated with factors that increase intracolonic pressure, especially diet and exercise. For example, diets low in fiber but abundant in red meat increase the risk of diverticular disease [1,4]. Other risk factors include age, lack of exercise, smoking, obesity, and opioid use [1,4].

Disease severity has been shown to increase with the severity of some patient risk factors. For example, patients’ body mass index (BMI) has been demonstrated to have a positive correlation with the risk for diverticulitis and resultant bleeding [6]. Furthermore, previous and current opioid use has been shown to increase not only the risk of diverticulitis but also severe complications of the disease, including perforation [7-10].

Opioids bind to G protein-coupled receptors, which, upon interaction with a ligand, close presynaptic calcium channels, and open postsynaptic potassium channels, thereby resulting in a reduction in neurotransmitter release and neuronal hyperpolarization respectively [11]. In effect, opioids reduce neuronal excitability via their actions on both pre- and post-synaptic neuronal endplates within synapses [11,12]. Opioid receptors, especially µ-receptors, are distributed widely throughout the GI tract. Found within myenteric and submucosal plexuses, they affect not only smooth muscle contraction but also neurohormonal signaling [11,12]. Through inhibition of neurotransmitter release throughout the GI tract, opiates result in decreased colonic motility, resulting in slow stool transit, ultimately increasing intraluminal pressures. Morphine specifically has been demonstrated to decrease colonic motility and increase smooth muscle tone, thereby resulting in constipation [13].

While a link between opioid use and diverticulitis, including perforation as a complication, has been established, few studies have examined the link between opioids and other complications of diverticulitis that also carry significant mortality and morbidity. Since narcotic use has been shown to increase the risk of perforation, likely as a result of increased intraluminal pressure, other complications of diverticulitis, including bleeding, sepsis, abscess formation, fistula formation, and obstruction, could also be affected by opioid use. Therefore, studying a possible correlation is worthwhile in order to better qualify the risks that patients exposed to opioid analgesics or those with a history of opioid abuse carry when admitted for diverticulitis. Our goal is to explore this possible correlation by utilizing the data in the National Inpatient Sample (NIS).

## Materials and methods

Data/source population 

The NIS was utilized to examine the data of patients admitted between 2008 and 2014. The International Classification of Diseases, 9th Revision (ICD-9) codes were utilized to select patients aged 18 years or older with a primary diagnosis of acute diverticulitis. The experimental group consisted of patients hospitalized with acute diverticulitis and a secondary diagnosis of opioid use disorder, opioid dependence, or opioid poisoning. Exclusion criteria included patients with a diagnosis of opioid use disorder in remission.

Statistical analysis

Categorical variables were analyzed via Chi-square analysis and continuous variables were analyzed via student T-tests. Elixhauser Comorbidity Index (ECI) scores were calculated based on weighted scores from 29 different comorbidities, enabling the prediction of overall mortality and readmission rates for both groups. ECIs were generated via SAS On-demand for Academics (SAS Institute Inc., Cary, NC) and IBM SPSS Statistics version 28.0.0 (IBM Corp., Armonk, NY), and these scores were compared across both groups via univariate analyses. Univariate and multivariate analyses were used to generate odds ratios (OR). Multivariate analyses using binary logistic regression were performed with inpatient mortality as the primary outcome. Other studied outcomes included perforation, bleeding, sepsis events, ileus, abscess formation, obstruction, and fistula formation. Length of hospital stay and total inpatient costs were also examined. Multivariate analysis factoring in age, sex, ethnicity, income status, and ECI scores was used to characterize and assess the effects of opioid use on mortality and secondary outcomes.

## Results

Patients with pre-existing opioid use were on average younger than their counterparts (54.47 versus 59.57 years) (Table 1). While the majority in both groups were female, relatively fewer females were found in the opioid use group (51.1% versus 57.6%) (Table 1). Most patients in both groups were Caucasian (74.5% in the opioid group versus 77.4% in the non-opioid group); however, the second most common race in the opioid group was black (11.1% versus 8%) while that in the non-opioid use group was Hispanic (10.7% versus 10.6%) (Table 1).

**Table 1:** Patient demographics SD: standard deviation

Variables		No opioid use (n=1,517,088)	Opioid use (n=2,980)
Age, years, mean (SD)		59.57 (15.58)	54.47 (13.69)
Sex, n (%)	Male	642,185 (42.4)	1,457 (48.9)
	Female	873,257 (57.6)	1,523 (51.1)
Race, n (%)	Caucasian	1,064,487 (77.4)	2,104 (74.5)
	Black	109,553 (8.0)	314 (11.1)
	Hispanic	146,947 (10.7)	299 (10.6)
	Asian/Pacific Islander	12,354 (0.9)	5 (0.2)
	Native American	6,210 (0.5)	31 (1.1)
	White	35,506 (2.6)	70 (2.5)

Patients with pre-existing opioid use hospitalized for acute diverticulitis had similar mortality rates to their counterparts (0.5% versus 0.5%) (Table 2). Those with a history of opioid use had higher rates of bleeding (1.5% versus 0.7%), sepsis (2.7% versus 1.5%), and obstruction (2.3% versus 1.5%) (Table 2). On the other hand, those without pre-existing opioid use had higher rates of perforation (0.7% versus 0.5%), fistula formation (0.7% versus 0.3%), abscess formation (14.4% versus 12.3%), and ileus (5.4% versus 5.1%); however, rates of ileus and perforation were statistically insignificant (Table 2).

**Table 2:** Rates of mortality and patient complications CI: confidence interval

	No opioid use, n (%)	Opioid use, n (%)	Odds ratio (95% CI)	P-value
Deceased	7,577 (0.5)	15 (0.5)	1.008 (0.607-1.675)	0.976
Perforation	10,107 (0.7)	15 (0.5)	0.755 (0.454-1.254)	0.276
Fistula	10,664 (0.7)	10 (0.3)	0.476 (0.26-0.89)	<0.01
Bleed	9,997 (0.7)	46 (1.5)	2.364 (1.766-3.165)	<0.01
Sepsis event	23,132 (1.5)	81 (2.7)	1.806 (1.447-2.252)	<0.01
Abscess	219,152 (14.4)	367 (12.3)	0.832 (0.746-0.929)	<0.01
Ileus	81,393 (5.4)	152 (5.1)	0.949 (0.81-1.12)	0.527
Obstruction	22,864 (1.5)	69 (2.3)	1.55 (1.22-1.97)	<0.01

ECI readmission rates (26.52 versus 8.43), length of stay (5.89 versus 4.87 days), and total charges ($44,568.2 versus $34,130.37) were higher in the opioid group (Table 3).

**Table 3:** Analysis of length of stay, total charges, and Elixhauser Indices SD: standard deviation

	No opioid use, mean (SD)	Opioid use, mean (SD)
Length of stay, days	4.87 (4.71)	5.89 (5.46)
Total charges, $	34,130.37 (48,275.80)	44,568.20 (65,743.33)
Elixhauser mortality	2.75 (7.28)	-3.65 (7.985)
Elixhauser readmit	8.43 (11.32)	26.52 (12.66)

Multivariate analysis demonstrated a significant effect in terms of sex [OR: 0.946, 95% confidence interval (CI): 0.897-0.998], Hispanic race (OR: 0.695, 95% CI: 0.621-0.779), Asian or Pacific Islander race (OR: 0.496, 95% CI: 0.353-0.698), and Native American race (OR: 1.529, 95% CI: 1.105-2.118) (Table 4).

**Table 4:** Odds ratios generated from multivariate analysis CI: confidence interval

	Odds ratio	95% CI lower	95% CI upper
Age	1.06	1.058	1.062
Female	0.946	0.897	0.998
Black	0.929	0.838	1.03
Hispanic	0.695	0.621	0.779
Asian or Pacific Islander	0.496	0.353	0.698
Native American	1.529	1.105	2.118
Other race	0.923	0.774	1.101

## Discussion

Opioids act on multiple organ systems, especially the alimentary tract, due to a high preponderance of opioid receptors in the GI system [11-13]. They can result in gastroesophageal reflux, constipation, the passage of hard stools, abdominal cramping, as well as a sensation of bloating and incomplete defecation [14,15]. Not only can they induce new gastrointestinal disorders, but they can also exacerbate pre-existing GI pathologies, including diverticular disease [7-10]. Increased intracolonic pressure is the key pathophysiologic mechanism of diverticular formation. Therefore, factors that increase intraluminal pressure would not only precipitate the formation of diverticula but also exacerbate the existing disease, increasing the incidence of diverticulitis and its complications, including perforation, bleeding, superimposed infection, and fistula formation.

In this study, we found that patients hospitalized for acute diverticulitis with a history of opioid use (diagnosed as either opioid use disorder, opioid dependence, or opioid poisoning) had higher rates of bleeding, sepsis, abscess formation, bowel obstruction, and fistula formation than patients without a history of opioid use. Bleeding in diverticular disease is due to injury to perforating vessels of the vasa recta, which can occur as a result of increased pressure [8,10]. Increased rates of diverticular bleeding have been demonstrated in opioid use; however, few studies have examined the rates of bleeding in diverticulitis specifically. Despite this, the mechanism behind bleeding in both cases is likely the same: slowed colonic transit as a result of narcotic use resulting in increased pressure and damage to perforating nutrient arteries crossing the colonic wall [8,10,16].

Rates of bowel obstruction were also elevated in opioid-using patients, likely secondary to opioid-induced inhibition of colonic contractility and mucosal secretions [17]. Specifically, opioid receptor agonists suppress the activation of post-synaptic motor neurons responsible for smooth muscle contraction, thereby interrupting the contractile phase of peristalsis, resulting in colonic stasis [17,18]. Furthermore, opioids prevent the activation of secretomotor neurons, thereby inhibiting luminal mucus release, which normally softens stool consistency, resulting in dry, hard stool [17-19]. In summation, opioids inhibit colonic motility and mucous secretion, resulting in decreased transit and formation of hard stool, in effect causing ileus and constipation. Strangely, rates of perforation were lower in patients with a history of opioid use (without statistical significance), despite multiple previous studies demonstrating the opposite [7-10].

Rates of sepsis were higher in the opioid use group, but rates of abscess formation were lower, which is a perplexing finding. Firstly, intra-abdominal abscesses are most commonly caused by perforation, and resultant peritoneal seeding by intracolonic bacteria [20]. The increased risk of perforation from opioids, as discussed previously, should therefore predispose the patient to an increased risk of abscess and sepsis as well [20,21]. However, in our study, opioid use was associated with decreased rates of fistula formation. The pathophysiology of fistula formation in diverticulitis involves either extension of a pre-formed inflamed diverticulum adjacent to another hollow viscus, or the erosion of an abscess into the hollow organ resulting in the formation of an epithelialized track between them [22,23]. Therefore the correlation we discovered between opioid use and the incidence of colonic fistulae and abscesses does not match our expectations, as we believed that there would be a positive relationship between these complications and opioid use. This disparity likely stems from our finding that perforation rates were lower in opioid-using patients, which contradicts previously established data.

This study has a few limitations, including the fact that patients with a history of opioid abuse are more likely to be immunocompromised than the general population, as this population is at increased risk for HIV infection, endocarditis, and other severe infections [24,25]. Therefore, patients with a history of opioid use have other risk factors that may predispose them to serious infections, sepsis, and abscess formation. This study as a whole is limited by its reliance on the NIS database alone, which uses ICD-9 codes to identify specific diagnoses of interest. Patients with a diagnosis that was not coded for during their hospital stay may not be represented in the sample, and hence the numbers obtained from the database may not be representative of the general population. In effect, not all patients with a history of opioid use disorder may have been identified, thereby skewing the data to produce the unexpected results we found.

Our analysis, in effect, demonstrates that patients with a diagnosis of opioid use disorder are at significantly higher risk for complications of diverticulitis, including sepsis, bleeding, and bowel obstruction. Therefore, physicians taking care of patients with a history of opioid abuse should be cognizant of the narcotic’s widespread systemic effects. Gastroenterologists in particular must be aware of the ubiquity of opioid receptors throughout the alimentary canal, and opioid use’s effects on the secretory and contractile capabilities of the GI tract.

## Conclusions

Opioid use disorder has been associated with an increased risk for diverticular disease and certain complications, including perforation. Our study further elaborates on this correlation and demonstrates that these patients are also at increased risk of other complications of diverticulitis with serious mortality and morbidity, including bleeding and sepsis. Past illicit drug use, especially opioids, must be identified by the physician in all patients, as opioid receptors are ubiquitous and the use of this narcotic may affect multiple organ systems. Specifically, gastroenterologists must be cognizant of patients with a history of opioid abuse in order to better risk-stratify those with diverticulitis and to more readily recognize complications of the disease when they arise.
